# Adolescent Cocreation in Digital Health: From Passive Subjects to Active Stakeholders

**DOI:** 10.2196/70020

**Published:** 2025-02-20

**Authors:** Alina Yang

**Affiliations:** 1 Scarsdale High School Scarsdale, NY United States

**Keywords:** adolescent health, digital health literacy, adolescents, online health information, co-design, health education, eHealth literacy, social media

Dear Editor,

The research publication entitled “Developing an Educational Resource Aimed at Improving Adolescent Digital Health Literacy: Using Co-Design as Research Methodology” [1] represents a pivotal intervention in the domain of youth-centric health research. With co-design effectively decentralizing traditional research frameworks by considering authentic adolescent perspectives on digital health literacy through their critiques of accessibility, personal and social factors, and information overload, resulting educational resources were able to incorporate more engaging, culturally and generationally relevant content to improve adolescents’ digital health literacy skills and self-efficacy.

Of all studies involving individuals aged 12-18 years, less than 1% reported using youth advice, and barriers to youth participation included limited researcher understanding, lack of resources, and organizational challenges [2]. This alarming underrepresentation epitomizes a systemic failure to acknowledge adolescents as active contributors to the knowledge that directly impacts their lives, instead perpetuating a research production hierarchy that undermines the unique cognitive capabilities and viewpoints of youth populations. While a variety of innovative digital health interventions, such as social networking sites, apps, and video games, have been developed to support youth, the successful development of these tools requires consistent input from target users [3]. Only in this way can we ensure the alignment of protocols, programs, and interventions to adolescent criteria of trustworthiness, safety, peer-to-peer connection, quality, and engagement, as well as the related usability and accessibility of tools among adolescents.

Research on our youth should ultimately be conducted by our youth, but more importantly, with our youth. Adolescents should not be arbitrarily labeled as mere resources for data but rather included in a detailed and thorough knowledge generation apparatus, including both study design and program implementation [4]. Promisingly, youth participatory action research fosters youth leadership in identifying health values and actions while balancing health promotion with personal growth and development [5]. This approach allies a commitment to empowering youth as cocreators with the recognition that their lived experiences are essential in designing interventions that truly resonate with their generation’s needs, particularly in a digital age where adolescents serve as primary consumers and innovators of social media and technology, respectively.

In traditional frameworks, adolescents are often regarded as passive subjects, with their needs, preferences, and involvement dictated by researchers, funders, or institutional mandates. But when youth are positioned as equals, the collaboration becomes more genuine, and the resulting health tools are more likely to meet their evolving demands. This shift is critical, particularly in digital health, where trends, technologies, and social norms change constantly, and youth are often the most proficient at staying ahead of these shifts. Thus, interventions, when co-designed with the very individuals they seek to serve, will be inherently more attuned to their preferences, technological fluency, and social contexts, thereby maximizing their potential for adoption, use, and sustainable impact. A radical restructuring of adolescent research involvement is long overdue, and we must redefine the processes by which youth health and knowledge are generated, validated, and disseminated to not just meet but anticipate their needs and realities.
